# Effects of Deoxycholylglycine, a Conjugated Secondary Bile Acid, on Myogenic Tone and Agonist-Induced Contraction in Rat Resistance Arteries

**DOI:** 10.1371/journal.pone.0032006

**Published:** 2012-02-16

**Authors:** Sandeep Khurana, Hema Raina, Valeria Pappas, Jean-Pierre Raufman, Thomas L. Pallone

**Affiliations:** 1 Division of Gastroenterology and Hepatology, VA Maryland Health Care System and University of Maryland School of Medicine, Baltimore, Maryland, United States of America; 2 Department of Physiology, University of Maryland School of Medicine, Baltimore, Maryland, United States of America; 3 Division of Nephrology, Division of Nephrology, Baltimore, Maryland, United States of America; Centre of Marine Sciences & University of Algarve, Portugal

## Abstract

**Background:**

Bile acids (BAs) regulate cardiovascular function via diverse mechanisms. Although in both health and disease serum glycine-conjugated BAs are more abundant than taurine-conjugated BAs, their effects on myogenic tone (MT), a key determinant of systemic vascular resistance (SVR), have not been examined.

**Methodology/Principal Findings:**

Fourth-order mesenteric arteries (170–250 µm) isolated from Sprague-Dawley rats were pressurized at 70 mmHg and allowed to develop spontaneous constriction, i.e., MT. Deoxycholylglycine (DCG; 0.1–100 µM), a glycine-conjugated major secondary BA, induced reversible, concentration-dependent reduction of MT that was similar in endothelium-intact and -denuded arteries. DCG reduced the myogenic response to stepwise increase in pressure (20 to 100 mmHg). Neither atropine nor the combination of L-NAME (a NOS inhibitor) plus indomethacin altered DCG-mediated reduction of MT. K^+^ channel blockade with glibenclamide (K_ATP_), 4-aminopyradine (K_V_), BaCl_2_ (K_IR_) or tetraethylammonium (TEA, K_Ca_) were also ineffective. In Fluo-2-loaded arteries, DCG markedly reduced vascular smooth muscle cell (VSM) Ca^2+^ fluorescence (∼50%). In arteries incubated with DCG, physiological salt solution (PSS) with high Ca^2+^ (4 mM) restored myogenic response. DCG reduced vascular tone and VSM cytoplasmic Ca^2+^ responses (∼50%) of phenylephrine (PE)- and Ang II-treated arteries, but did not affect KCl-induced vasoconstriction.

**Conclusion:**

In rat mesenteric resistance arteries DCG reduces pressure- and agonist-induced vasoconstriction and VSM cytoplasmic Ca^2+^ responses, independent of muscarinic receptor, NO or K^+^ channel activation. We conclude that BAs alter vasomotor responses, an effect favoring reduced SVR. These findings are likely pertinent to vascular dysfunction in cirrhosis and other conditions associated with elevated serum BAs.

## Introduction

Recent investigations expanded the physiological role of BAs beyond digestion and cholesterol metabolism to thyroid function, glucose metabolism and obesity [1]. Over the past decade, emerging evidence suggests that BAs also act as hormones with vasoactive properties [2]. BAs regulate cardiovascular function by interacting with plasma membrane receptors (TGR5, M_3_R and M_2_R), Big Ca^2+^-activated K^+^ channels (BK_Ca_) and nuclear receptors (FXR, PXR and VDR) [3]–[10]. They mediate vasodilation by mechanisms that are highly dependent on the arterial bed (central vs. peripheral) and type of BA (secondary vs. primary; amidated vs. unamidated) [11]–[13]. Moreover, whereas i*n vivo* infusion of high dose BAs reduces arterial blood pressure [11], a direct effect on systemic vascular resistance (SVR) has not been demonstrated. Small arteries (diameter ∼200 µm) are primary determinants of SVR; the arterial myogenic response, defined as vasoconstriction in response to increases in intraluminal pressure is critical for establishing SVR [14]. The splanchnic circulation is a major site of vascular resistance but effects of BAs on myogenic responses in splanchnic resistance arteries have not been investigated [15], [16].

BAs are amidated with glycine or taurine, thereby increasing solubility (see detailed reviews of BA metabolism in [2], [17]). Previously, we reported that conjugated BAs interact functionally with M_3_ subtype muscarinic receptors (M_3_R; encoded by *CHRM3*) [18]. Molecular modeling suggests that BAs share structural similarities with acetylcholine (ACh), a muscarinic receptor ligand; molecular surface structure and charge distribution on the taurine amide side chain closely resemble electrostatic charge distribution on ACh [5]. Using rodent aorta, we found that deoxycholyltaurine (DCT), the *taurine* conjugate of deoxycholic acid, reduces PE-induced tension, and in aorta isolated from *Chrm3^−/−^* mice, this response to DCT treatment is reduced [12]. A recent study in cardiomyocytes indicates that cholyltaurine interacts with M_2_R to mediate negative chronotropic effects [7]. Hence, in cardiovascular tissue, whereas a functional interaction between muscarinic receptors and *taurine*-conjugated BAs has been demonstrated a similar interaction for *glycine*-conjugated BAs has not been investigated.

Elevated serum BAs are proposed mediators of cardiovascular dysfunction in cirrhosis and intrahepatic cholestasis of pregnancy [19]–[22]. In both health and disease serum glycine-conjugated BAs are more abundant than taurine-conjugated BAs [20], [23], [24]. While the effects of taurine-conjugated BAs on cardiovascular function have been evaluated, neither the effects of BAs on MT, the key determinant of SVR, nor the effects of glycine-conjugated BAs on agonist-induced vasoconstriction have been examined [11], [25]. Hence, in the present study, we utilized 4^th^-order rat mesenteric arteries to compare the effects of ACh and deoxycholylglycine (DCG), the *glycine* conjugate of deoxycholic acid on pressure-induced vascular tone.

## Results

### Effect of DCG on myogenic tone in rat pressure-constricted fourth-order mesenteric arteries

**Figure 1:**
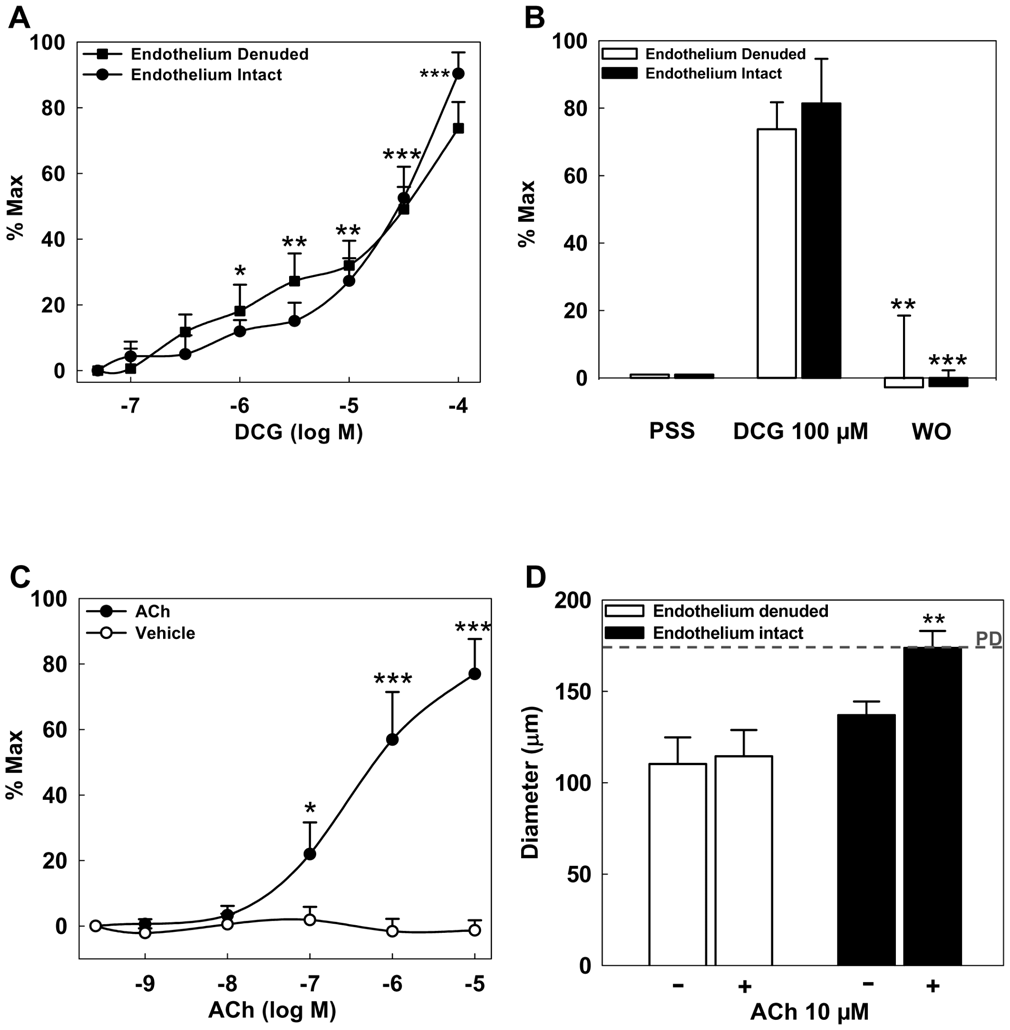
Effect of DCG and ACh on MT in rat 4^th^-order mesenteric arteries. (A) DCG induces concentration-dependent reduction of MT in endothelium-intact and -denuded arteries. Asterisks indicate significance when compared to the baseline for both preparations. (B) DCG (100 µM) reduced MT in endothelium-intact and -denuded arteries. This effect was reversed by washing with PSS; (WO-Wash out). Asterisks indicate significance when compared to DCG (100 µM)-mediated response in respective arterial preparations. (C) ACh induced concentration-dependent reduction of MT. There was no effect in vehicle-treated arteries. (D) Addition of 10 µM ACh had no effect on endothelium-denuded preparations while endothelium-intact preparation achieved diameters similar to passive diameter. Asterisks indicate significance when compared to untreated endothelium-intact arteries. The dashed-line represents passive diameter (PD). PD is the lumen diameter when VSM is completely inactive. (n = 4–5 arteries in each group).

To determine the effect of DCG on MT, increasing concentrations of DCG were added to pressure-constricted arteries. At 70 mmHg, spontaneous vasoconstriction, i.e. MT, was 31.87±2.29% of passive diameter (*P<0.01*). DCG induced concentration-dependent (0.1–100 µM) reduction of MT in endothelium-intact and -denuded preparations (Fig. 1A). In both, endothelium-intact and -denuded preparations, reduction of MT was readily reversed by washout (WO, Fig. 1B) with PSS. As a positive control, we verified that ACh induces concentration-dependent (0.001–10 µM) reduction of MT (Fig. 1C). Although, endothelium denudation did not alter DCG-mediated reduction of MT (Fig. 1A), it abolished ACh-induced reduction of MT (Fig. 1D). These data indicate that in pressurized fourth-order mesenteric rat arteries, DCG and ACh concentration-dependently reduce MT by different mechanisms. DCG-mediated reduction of MT is reversible and, unlike ACh, is endothelium-independent. These observations imply that the effects of DCG are not mediated by interaction with muscarinic receptors.

### Role of muscarinic receptors in DCG-induced reduction of MT in fourth-order mesenteric arteries

**Figure 2:**
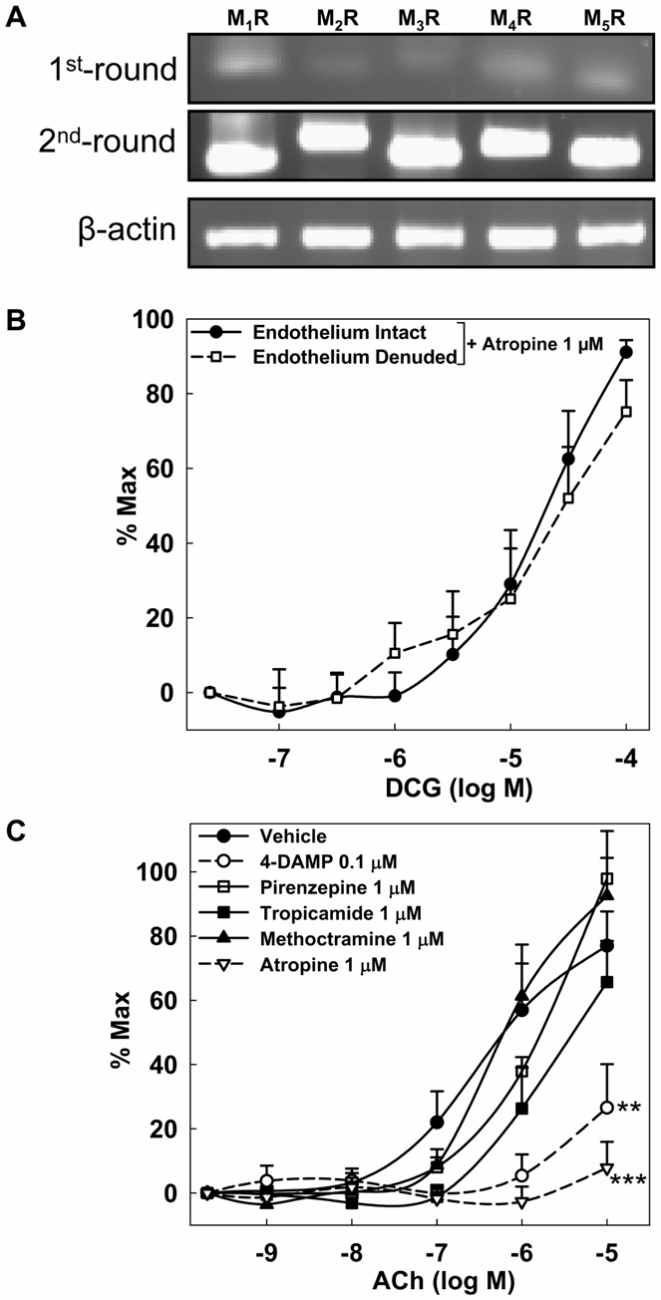
Role of muscarinic receptors in DCG- and ACh-induced reduction of MT in rat 4^th^-order mesenteric arteries. (A) PCR reveals expression of all muscarinic receptor subtypes (M_1_R-M_5_R). mRNAs were extracted from arteries for 3 rats. PCR products, obtained using primers shown in Table 1 (first round), were amplified for 30 cycles with the second-round primers (Table 2). Agarose gel electrophoresis of PCR products is shown. (B) DCG-induced concentration-dependent reduction of MT in endothelium-intact and -denuded arteries preincubated with 1 µM atropine. (C) 1 µM Atropine and 0.1 µM 4-DAMP markedly attenuated ACh-induced reduction of MT. Asterisks indicate significance when compared to vehicle-treated arteries. (n = 4–5 arteries in each group).

Previous studies of cardiovascular tissues indicate that BAs interact functionally with M_2_R and M_3_R [5], [7]. To determine the role of muscarinic receptors in DCG-induced reduction of MT, we first determined the expression profile of muscarinic receptor subtypes in rat fourth-order mesenteric arteries. As shown in Figure 2A, using PCR with mRNA extracted from rat fourth-order mesenteric arteries, we found that mRNA for all muscarinic receptor subtypes is expressed. Since DCG reduced MT in endothelium-intact and -denuded arteries (Fig.1A), we evaluated the ability of the nonselective MR blocker, atropine, to inhibit DCG-induced reversal of MT. As shown in Figures 2B and 2C, whereas pre-incubation with atropine prevented ACh-mediated reduction of MT, DCG-mediated reduction of MT was not altered. To determine the muscarinic receptor subtype(s) responsible for ACh-induced vasodilation, subtype-selective inhibitors were tested. Pirenzepine (M_1_R blocker) and methoctramine (M_2_R blocker) did not alter ACh-induced reduction of MT (Fig. 2C). Tropicamide (M_3,4_R blocker) marginally shifted the ACh response curve to the right while 4-DAMP (M_3_R blocker) markedly attenuated the response (Fig. 2C). However, 4-DAMP had no effect on myogenic response (Fig. S1). Collectively, these data indicate that in rat fourth-order mesenteric arteries: 1) mRNA for all MR subtypes is expressed, 2) DCG-induced reduction of MT is muscarinic receptor-independent and 3) M_3_R mediates ligand (ACh)-induced reduction of MT.

### Role of NO and K^+^ channels in DCG-induced reduction of MT in fourth-order mesenteric arteries

**Figure 3:**
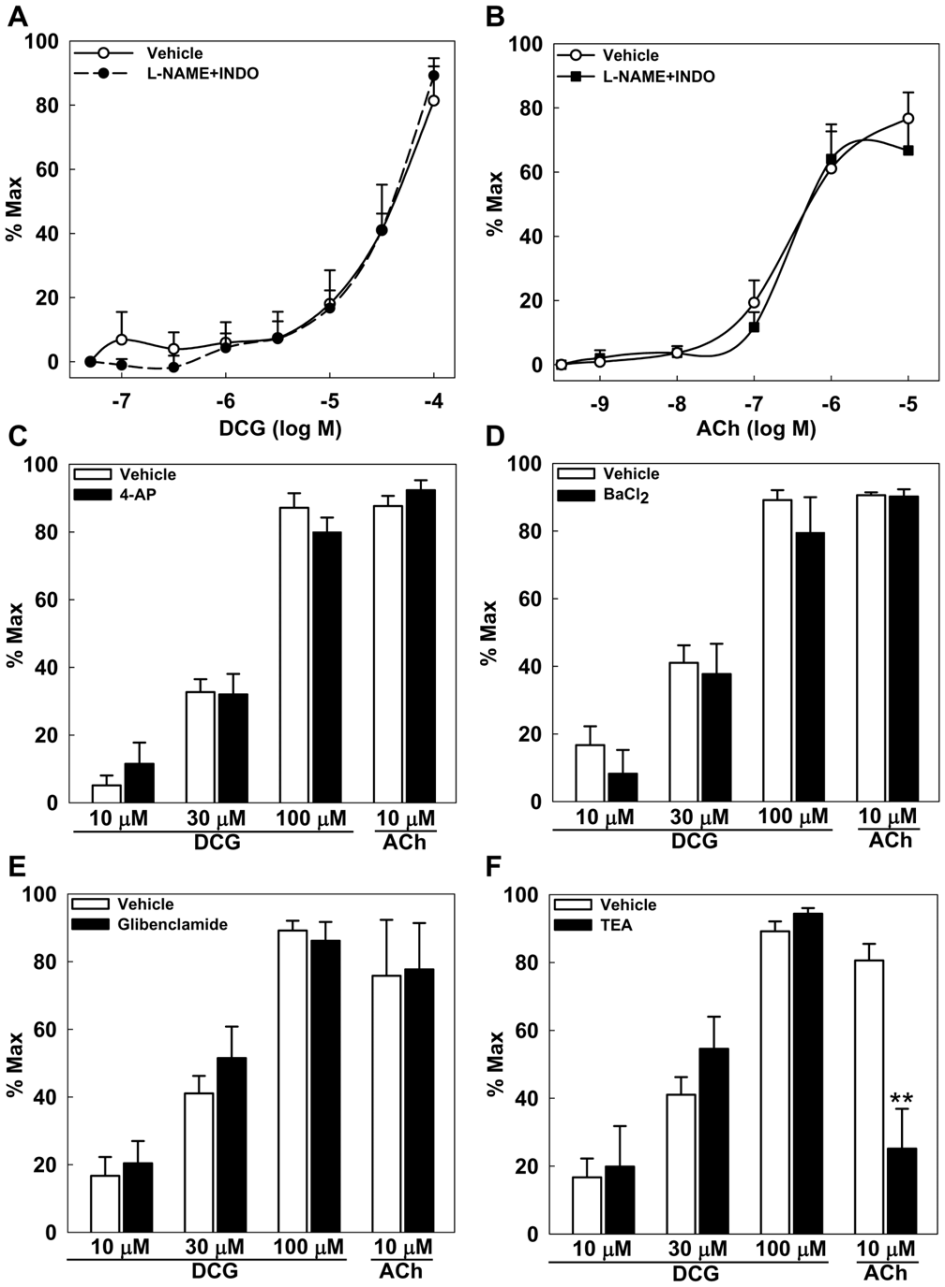
Role of NO and K^+^ channels in DCG- and ACh-induced reduction of MT in rat 4^th^-order mesenteric arteries. (A) DCG induced concentration-dependent reduction of MT in arteries incubated with L-NAME (300 µM) and INDO (10 µM). (B) ACh induced concentration-dependent reduction of MT in arteries incubated with L-NAME and INDO. (C–F) Addition of 4-aminopyradine (K_V_ blocker, 1 mM), BaCl_2_ (K_IR_ blocker, 30 µM), glibenclamide (K_ATP_ blocker, 10 µM) or TEA (K_Ca_ blocker, 10 mM) had no effect on DCG-mediated reduction of MT. Only TEA attenuated ACh-mediated reduction of MT; all experiments were conducted in the presence of L-NAME and INDO. Asterisks indicate significance when compared to vehicle-treated arteries. (n = 4–5 arteries in each group).

NO plays a major role in MT of resistance arteries. Based on the results in Figure 1A, endothelium-derived NO is unlikely to play a role in DCG-induced reduction of MT. Nonetheless, NO derived from nNOS, expressed in VSM or periarterial nerves, remains a possible mediator [26]. To exclude this, the effects of DCG and ACh on MT were determined in arteries incubated with the global NOS isoform inhibitor, L-NAME. To exclude a role for byproducts of cyclooxygenase activity, indomethacin (INDO) was used. As shown in Figure 3A and 3B, both DCG and ACh induced concentration-dependent reduction of MT in arteries incubated with L-NAME plus INDO excluding a role for NO and prostaglandins. Additionally, inhibition of guanylyl cyclase, the NO target in VSM cells, had no effect on DCG-induced reduction of MT (Fig. S2). These findings suggested vasodilation via an endothelium-derived hyperpolarization factor (EDHF)-dependent mechanism.

In resistance arteries, K^+^ channels play a major role in EDHF-dependent responses and VSM cells express all major types of K^+^ channels that regulate MT [27]–[30]. To test for a role of K^+^ channel activation in DCG-mediated reduction of MT, selective inhibitors of voltage-gated (K_V_), inward rectifier (K_IR_), ATP-dependent (K_ATP_) and Ca^2+^-activated K^+^ (K_Ca_) channels were used. As shown in Figure 3C–F, neither 4-AP (K_V_ blocker1 mM), BaCl_2_ (K_IR_ blocker, 30 µM), glibenclamide (K_ATP_ blocker, 10 µM) nor TEA (K_Ca_ blocker, 10 mM) prevented DCG-induced reduction of MT. In contrast, TEA attenuated the ACh-induced reduction of MT, supporting the ACh-stimulated generation of EDHF. Similarly, charybdotoxin (ChTX 50 nM, IK_Ca_ blocker) plus apamin (50 nM, SK_Ca_ blocker) abolished ACh-induced reduction of MT (data not shown). All experiments were conducted in the presence of L-NAME and INDO. These data indicate that DCG-induced reduction of MT is independent of VSM K^+^ channels whereas ACh-induced reduction of MT is K_Ca_–dependent.

Based on these findings, we concluded that in small resistance arteries, DCG reduces MT independent of muscarinic receptor activation, and NO- or EDHF-induced modulation of K^+^ channel activity. In view of this, we hypothesized that DCG acts by directly or indirectly activating VSM Ca^2+^ entry pathways that generate and maintain vascular tone [14]. To test this hypothesis, we evaluated the effect of DCG on myogenic response, KCl- and agonist-induced vasoconstriction.

### Effect of DCG on myogenic response in fourth-order mesenteric arteries

**Figure 4:**
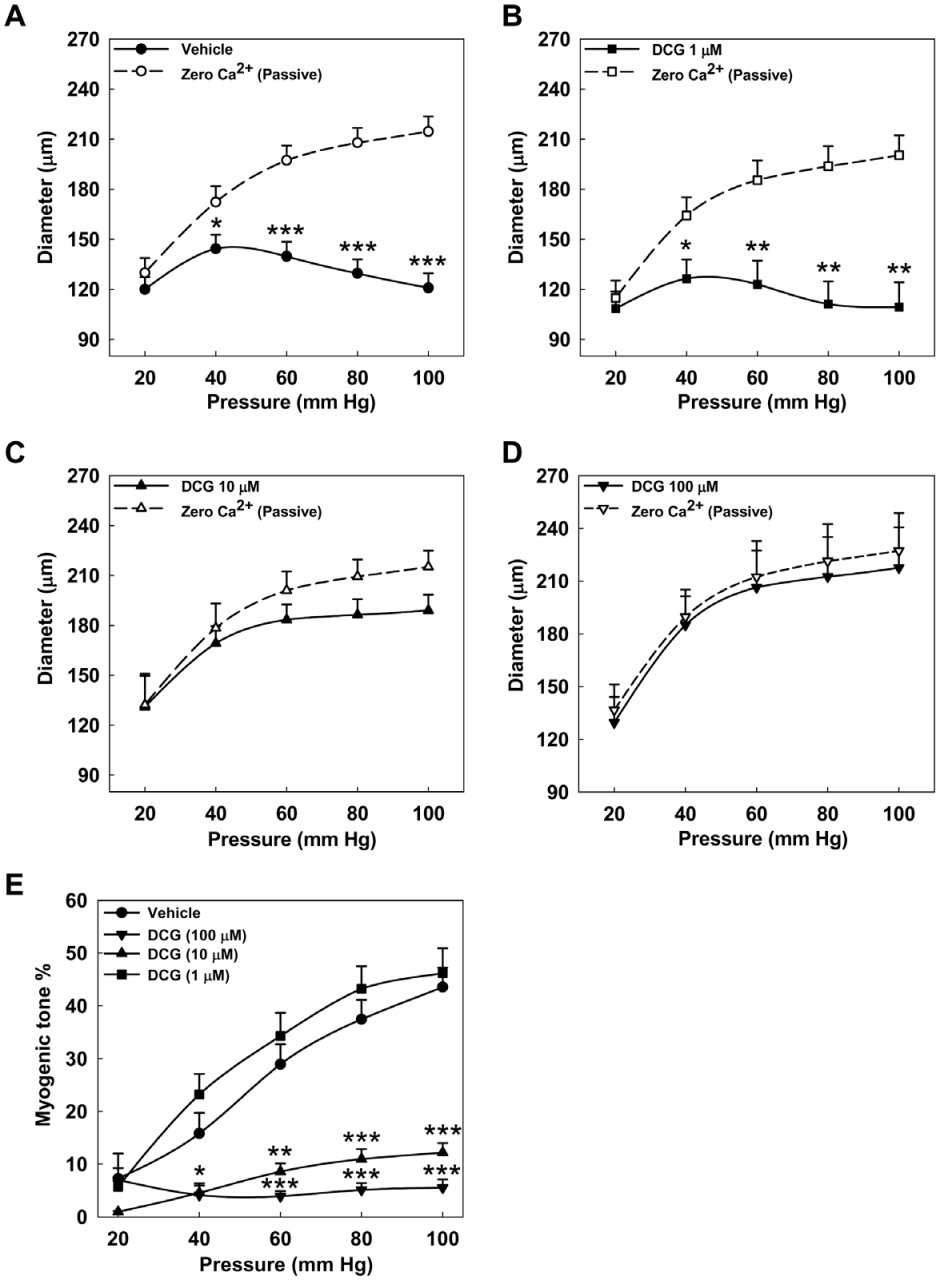
Effect of DCG on myogenic response. (A–D) Fourth-order rat mesenteric arteries pretreated with vehicle and DCG (1, 10, 100 µM) were subjected to a series of intraluminal pressure steps between 20 and 100 mmHg and spontaneous tone was allowed to develop until a stable diameter was achieved (solid lines). The pressure-response was repeated in Ca^2+^-free physiological salt solution (PSS) with 3 mM EGTA and 0.01 mM diltiazem (dashed lines). Asterisks indicate significant differences between the diameters at each pressure step. (E) Summary data. MT was calculated as the percent difference in diameter observed for Ca^2+^-containing vs. Ca^2+^-free PSS at each pressure. DCG (10 and 100 µM) prevented development of MT in small mesenteric arteries. Asterisks indicate significance when compared to vehicle-treated arteries. (n = 5–7 arteries in each group).

Increase in transmural pressure induces constriction of resistance arteries, the myogenic response. To test whether DCG can inhibit pressure induced contraction, fourth-order resistance arteries were subjected to stepwise increases of intravascular pressure from 20 to 100 mmHg in 20-mmHg increments in the presence of vehicle or 1, 10 or 100 µM DCG. In vehicle-treated arteries, increases in pressure induced a biphasic response consisting of an increase followed by reduction in diameter (Fig. 4A). In arteries treated with 1 µM DCG, myogenic response was similar to vehicle (Fig. 4B). However, with 10 µM DCG, the myogenic response was blunted (Fig. 4C) and with 100 µM DCG, it was absent (Fig. 4D). The calculated MT for each pressure measurement is summarized in Figure 4E. Collectively, these data indicate that DCG can markedly suppress myogenic contraction of mesenteric arteries.

### Effect of DCG on vascular smooth muscle (VSM) Ca^2+^ in fourth-order mesenteric arteries

**Figure 5:**
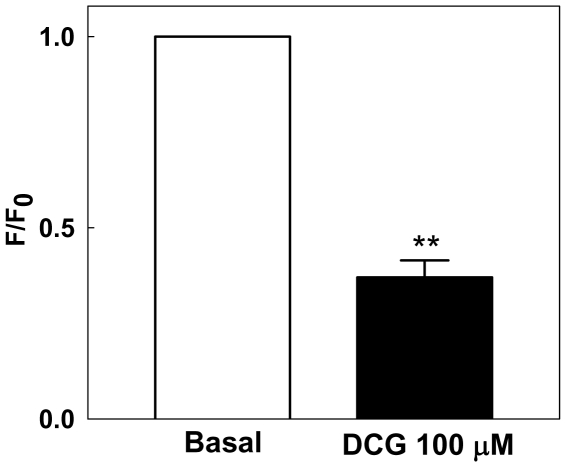
DCG reduces VSM Ca^2+^ in 4^th^-order mesenteric arteries from rats with MT. Ca^2+^ fluorescence was measured in Fluo-2-loaded arteries before and after incubating with DCG 100 µM for 5 minutes. DCG reduced the arterial VSM Ca^2+^ fluorescence by ∼50%. (n = 3 arteries in each group).

To test the hypothesis that DCG acts by inhibiting VSM Ca^2+^ signaling, we investigated its effects on intracellular cytoplasmic Ca^2+^ concentration in arteries loaded with Fluo-2. Fluorescence was measured before and after incubating arteries with 100 µM DCG for 5 min. As shown in Figure 5, DCG reversibly reduced VSM Ca^2+^ fluorescence to approximately 50% of baseline. These data support the hypothesis that reduction of cytoplasmic Ca^2+^ concentration underlies the ability of DCG to reduce MT.

### Role of high-dose Ca^2+^on DCG-induced reduction of myogenic response

**Figure 6:**
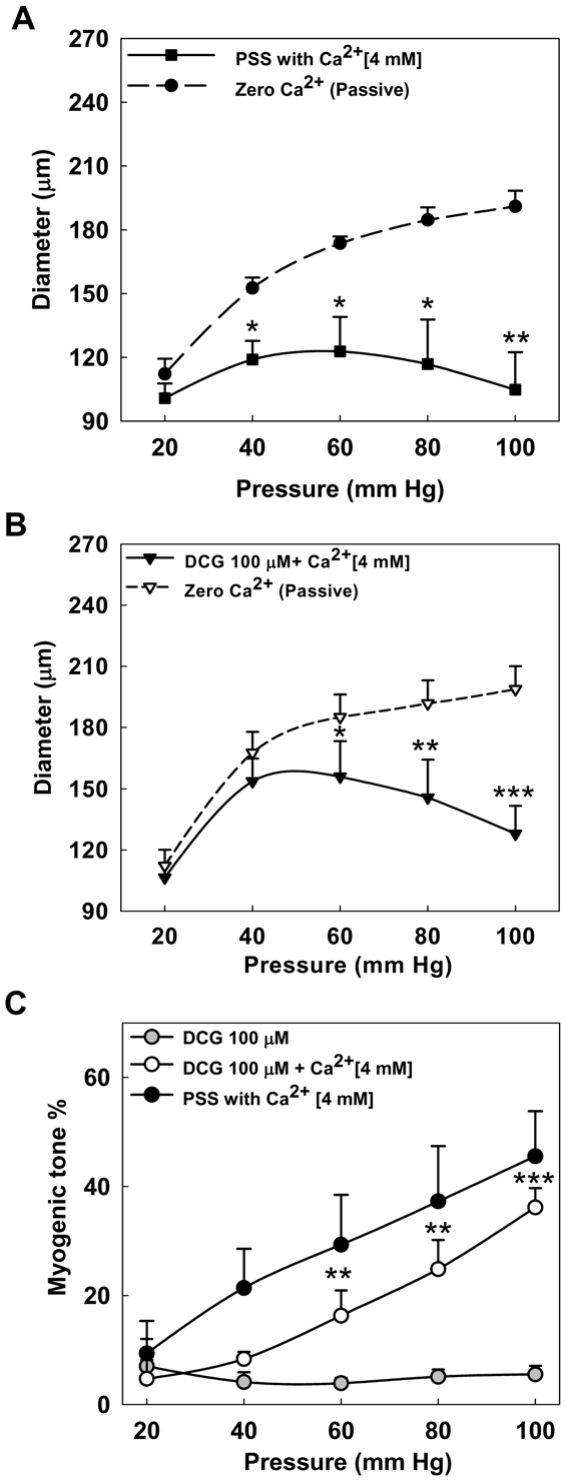
Effect Ca^2+^ (4 mM)-PSS on DCG-induced reduction of myogenic response. (A, B) To assess myogenic response, rat fourth-order mesenteric arteries were subjected to a series of intraluminal pressure steps between 20 and 100 mmHg in Ca^2+^(4 mM)-PSS alone or with 100 µM DCG. Spontaneous tone was allowed to develop until a stable diameter was achieved (solid line). The pressure-response was repeated in Ca^2+^-free PSS with 3 mM EGTA and 0.01 mM diltiazem (dashed line). Asterisks indicate significant differences between the diameters at each pressure step. (C) Summary data. MT was calculated as the percent difference in diameter observed for Ca^2+^ (4 mM)-containing vs. Ca^2+^-free PSS at each pressure. The bottom myogenic response curve for DCG (100 µM) in normal PSS is derived from Figure 4. Incubation of arteries with DCG (100 µM) in Ca^2+^ (4 mM)-PSS attenuated the DCG-induced reduction of myogenic response. Asterisks indicate significant difference in MT for the respective pressure step when compared to arteries incubated with DCG (100 µM) in normal PSS. (n = 4–7 arteries in each group).

We showed above that DCG reduces myogenic response and VSM Ca^2+^ in pressurized arteries. Based on these findings we hypothesized that raising extracellular Ca^2+^ concentration to promote VSM Ca^2+^ entry would prevent DCG-induced reduction of MT. Myogenic response was determined in the presence of 100 µM DCG with high Ca^2+^ (4 mM)-PSS [31]. As shown in Figure 6A, in high Ca^2+^ (4 mM)-PSS, pressure increases induced an initial increase followed by reduction in diameter, similar to normal PSS (Fig. 4A). In contrast to the ability of DCG to inhibit myogenic contraction in PSS (2 mM Ca^2+^, see Fig. 4D, 4E), high extracellular Ca^2+^ (4 mM) rendered it much less effective (Fig. 6B). The calculated MT for luminal pressure increments is summarized in Figure 6C, wherein the myogenic response in normal PSS is also reproduced from data in Figure 4. These data support a role for modification of VSM cytoplasmic Ca^2+^ signaling in DCG-induced reduction of MT.

### Effect of DCG on agonist-induced vasoconstriction

**Figure 7:**
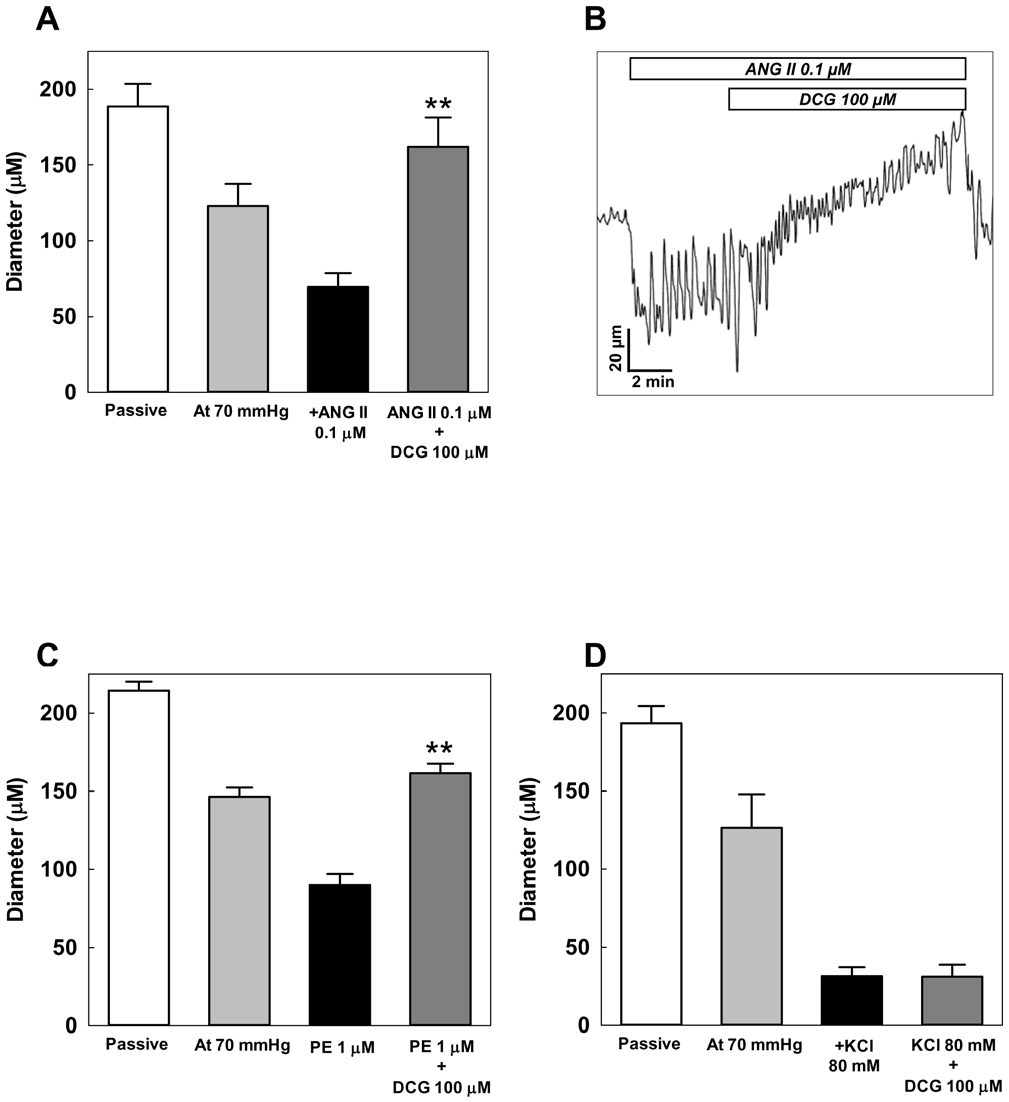
Effect of DCG on agonist-induced vasoconstriction. (A) At 70 mmHg, the arteries developed spontaneous vasoconstriction that was further augmented by adding ANG II (0.1 µM). DCG attenuated ANG II-induced vasoconstriction. (n = 5 arteries). (B) An example tracing of the effect of DCG on ANG II-induced vasoconstriction. (C) At 70 mmHg, the arteries developed spontaneous vasoconstriction that was further augmented by adding PE (1 µM). DCG attenuated PE-induced vasoconstriction. (n = 12 arteries). (D) DCG (100 µM) did not alter 80 mM KCl-induced vasoconstriction. (n = 3 arteries). Asterisks indicate significant differences when compared to ANG II and PE alone.

To determine its effect on agonist-induced VSM contraction, DCG was applied following Angiotensin II (ANG II)-, PE- and KCl-induced vasoconstriction. Arteries with pre-existing MT were further constricted by ANG II (0.1 µM) an effect reversed by DCG (Fig. 7A). A representative tracing of DCG-induced reversal of ANG II effect is shown in Figure 7B. DCG also attenuated PE (1 µM)-induced vasoconstriction (Fig. 7C). In contrast, DCG did not alter KCl (80 mM)-induced vasoconstriction (Fig. 7D). These results indicate that DCG inhibits agonist-induced vasoconstriction, however, it has no effect on depolarization-mediated vasoconstriction.

### Effect of DCG on PE-induced Ca^2+^dynamics in fourth-order mesenteric artery VSM

**Figure 8:**
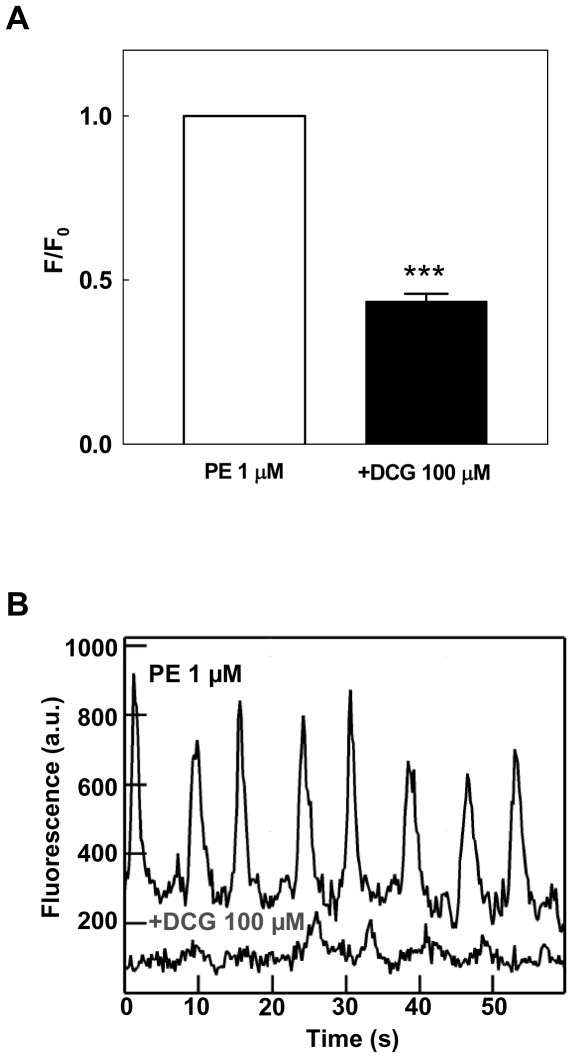
Effect of DCG on agonist-mediated changes in VSM Ca^2+^. (A) Fluo-2-loaded arteries were treated with PE 1 µM. Ca^2+^ fluorescence was measured before and after incubating with DCG 100 µM for 5 minutes. DCG reduced the arterial VSM Ca^2+^ fluorescence by ∼50%. (B) An example of fluorescence tracings from the same artery before and after addition of DCG is shown (a.u., arbitrary units). (n = 5 arteries in each group).

To test whether DCG can inhibit Ca^2+^ signaling that arises from adrenergic stimulation, we examined its effects in Fluo-2 loaded arteries treated with PE (1 µM). Fluorescence was measured before and after incubating arteries with 100 µM DCG for 5 min. As shown in Figure 8A, DCG blunted PE-induced Ca^2+^ signaling. Representative tracings of Ca^2+^ fluorescence before and after the addition of DCG are shown in Figure 8B. These data support the conclusion that DCG blunts adrenergic contraction, at least partially, by interfering with VSM Ca^2+^ signaling.

## Discussion

Under isobaric conditions, small resistance arteries develop partial constriction i.e., MT. These small resistance arteries constrict when subjected to increasing intraluminal pressure and dilate when the pressure is reduced, i.e. myogenic response [32]. Myogenic behavior (MT and myogenic response) governs local blood flow, systemic vascular resistance and blood pressure [14], [33], [34]. Hence, pressurized resistance arterial preparations are of value in the study of vasoactive properties of molecules pertinent to modulation of SVR [20], [22], [35], [36].

Our study identifies novel BA-mediated effects on vascular tone, which adds to the expanding profile of vasogenic properties of BAs. In rat resistance mesenteric arteries our data indicate that DCG, a *glycine*-conjugated secondary BA, reduces MT in an endothelium-independent and reversible manner. Although M3 muscarinic receptors play a dominant role in ACh- and DCT-[12], they do not contribute to DCG-mediated reduction of MT. Likewise, DCG-induced reduction of MT is NO- and prostaglandin-independent. Although K_Ca_ channel activation is important for ACh-induced reduction of MT, neither K_ATP_, K_V_, K_IR_ nor K_Ca_ activation plays a role in DCG-induced reduction of MT. DCG reduces basal and agonist-stimulated VSM intracellular [Ca^2+^], and a high-concentration of extracellular Ca^2+^ attenuates DCG-induced reduction of MT.

BAs interact functionally with muscarinic receptors [4], [5]. Previously, we showed that deoxycholyltaurine, the *taurine* conjugate of deoxycholic acid, induces vasodilation of PE-contracted rat and mouse aorta by M_3_R-dependent mechanisms [12]. A recent report indicated that in neonatal rat cardiomyocytes, taurocholate, the taurine conjugate of cholic acid, stimulates negative chronotropic effects by interaction with M_2_R; these actions were abolished by M_2_R knockdown [7]. Our experiments (Figs. 1 and 2) indicate that ACh reduces MT by interacting with M_3_R. Moreover, 4-DAMP, an M_3_R selective antagonist, did not alter on basal MT or myogenic response (Fig. S1), suggesting that the M_3_R-mediated responses are not constitutive and require activation by a ligand. Despite these findings and PCR verification of mesenteric expression of all muscarinic receptor subtypes, atropine did not block DCG-mediated reduction of MT, thereby showing that DCG is not a MR agonist. We did not examine the interaction of non-conjugated or taurine-conjugated DCA with MR. However, based our findings and those previously reported [7], [12] we speculate that amidation may be a determinant of BA interaction with vasoactive signaling pathways.

In small mesenteric arteries, both eNOS and nNOS contribute to the NO-mediated regulation of vascular tone [26]. There are conflicting reports about the role of NO in BA-mediated changes in vascular tone. In endothelial cell cultures, BAs activate NOS to generate NO [37], [38]. In rat and mouse aorta, DCT-induced vasodilation was attenuated by L-NAME [12]. In the present study, endothelial denudation had no effect on DCG-induced reduction of MT, excluding a role for eNOS. Vascular regulatory mechanisms are heterogeneous along the first- to fourth-order mesenteric arterial arcade [27]. Neither L-NAME, a non-selective NOS inhibitor, nor a combination of PGI_2_, NO and K^+^ channel blockers altered DCG-induced reduction of MT (Fig. 3C–3F). We confirmed expression of eNOS and nNOS and excluded a direct effect on guanyl cyclase by evaluating the effect of DCG in the presence ODQ (a guanyl cyclase inhibitor); ODQ did not block DCG-mediated reduction of MT (Fig. S2). These data provide novel evidence that DCG-induced reduction of MT is independent of NO or K^+^ channel activation.

VSM cells express various K^+^ channels whose activity can regulate arterial vascular tone. Dopico et al. demonstrated that selected BAs reversibly activate BK_Ca_ [6]. In cerebral arteries, lithocholic acid-induced, endothelium-independent vasodilation was blocked by iberiotoxin, a BK_Ca_ blocker. In our studies, TEA, an established non-selective K_Ca_ channel blocker [39], [40], had no effect on DCG- but significantly attenuated ACh-induced reduction of MT (Fig. 3F). Our findings regarding ACh are consistent with previous observations in small resistance arteries where EDHF plays a major role in ACh-induced reduction in MT [27]. However, our data contrast with studies that identify activation of BK_Ca_ as the sole mechanism of BA-induced vasodilation [13]. Factors that may account for differences between our findings and those of Bukiya et al [13] include: 1) use of arterial preparations from different arterial beds; mesenteric vs. cerebral; 2) use of dihydroxylated vs. monohydroxylated BAs; and 3) BA amidation – glycine-conjugated vs. native BA.

Discovery of BA-activated receptors including nuclear receptors such as FXR, VDR and PXR, and TGR5, a GPCR, have expanded investigations of cardiovascular effects of BAs [3], [41]–[43]. In the current study, we did not evaluate the role of nuclear receptors as they are transcription factors and therefore, unlikely to mediate immediate DCG-induced actions. Previously, TGR5 was shown to be expressed in hepatic sinusoidal endothelium that activates eNOS via cAMP-dependent mechanism [38], [44]. Whether TGR5 is expressed in mesenteric bed is not known, and in the current study, the lack of TGR5 antagonists prevented us from evaluating its role in DCG-mediated effects.

In VSM cells, G_q/11_-coupled receptors that induce vasoconstriction also function as membrane stretch sensors [45]. Our data indicate that DCG reduces both MT and agonist (PE-and ANG II)-induced vasoconstriction. Since G_q/11_-coupled receptors are common to pressure- and agonist-induced vasoconstriction, it is possible that DCG inhibits G_q/11_-dependent signaling to reduce vascular tone. DCG-induced reduction of MT and agonist-induced vasoconstriction appear to be Ca^2+^-dependent. Since DCG had no effect on high-dose KCl-induced vasoconstriction, it appears less likely to affect voltage-gated Ca^2+^channels. Regulation of VSM Ca^2+^ is complex, involves multiple ion channels and signaling proteins; knowledge in this area is continuously evolving. It is possible that DCG-induced inhibition of receptor-mediated activation of a Ca^2+^ entry pathway partially explains isolated DCG effects on Ang II- and PE- but not KCl-induced constriction. In future studies it may be appropriate to focus on the role of Ca^2+^ channels and Ca^2+^ sensitization in DCG-mediated effects on VSM Ca^2+^ and vasodilation.

Serum BA concentration in adult humans is ∼3 µM/l [20], [36]. In cirrhosis and obstructive jaundice, serum BA concentration can exceed >200 µM/l [19], [20], [36]. While the effects of taurine-conjugated BAs on vascular tone have been evaluated before, the effects of glycine-conjugated BAs have not been studied. In both, cirrhosis and obstructive jaundice, serum levels of *glycine*-conjugated BAs are elevated and are in the same range as those tested in this study [20]. We showed that a *glycine*-conjugated BA i.e. DCG, reduces pressure- and agonist-induced vascular tone by reducing VSM Ca^2+^. Cirrhosis and obstructive jaundice are characterized by decreased SVR and altered vascular tone, respectively [21], [46]. Since, small resistance arteries are important contributors to SVR, our findings may have biological and clinical importance with regard to hemodynamic changes associated with cirrhosis and obstructive jaundice. *In vivo* studies will be required to determine the effect of *glycine*-conjugated BAs on SVR.

## Materials and Methods

### Arterial isolation and cannulation

All experiments were performed according to protocols approved by the Institutional Animal Use and Care Committee of the University of Maryland School of Medicine (Protocol No: 0508008). Sprague-Dawley rats were housed in the animal facility with controlled temperature and lighting and allowed free access to water and a commercial rodent chow. Rats (100 to 150 g) were anesthetized by intraperitoneal injection of ketamine (80 mg/kg) and xylazine (10 mg/kg). The abdomen was opened and part of the distal mesenteric artery arcade was rapidly removed and transferred to a chamber containing ice-cold dissection solution containing (mM/l): 3.0 MOPS, 145.0 NaCl, 5.0 KCl, 2.5 CaCl_2_, 1.0 MgSO_4_, 1.0 KH_2_PO_4_, 0.02 EDTA, 2.0 sodium pyruvate and 5.0 glucose (pH 7.4). Small arteries [passive diameter (PD) = 170–260 µm] parallel to the small intestine, were dissected into 3–5-mm unbranched segments, cleaned and transferred to a perfusion chamber. The proximal end of vessels was cannulated with a glass micropipette (diameter: 100–150 µm), secured with a suture and blood was gently rinsed from the lumen. The distal end of the vessels was tied onto a second glass cannula. One cannula was connected to a servo-controlled pressure-regulating device (Living Systems; Burlington, VT), and the other to a closed stopcock. The chamber was transferred to an inverted microscope and the vessel superfused (4 ml/min) with PSS (mM/l): 112.0 NaCl, 25.7 NaHCO_3_, 4.9 KCl, 2.0 CaCl_2_, 1.2 MgSO_4_, 1.2 KHPO_4_, 11.5 glucose and 10.0 HEPES (pH 7.4, equilibrated with gas mixture: 5% CO_2_, 5% O_2_ and 90% N) [47]. Intraluminal pressure was slowly increased from 20 to 100 mmHg and vessels with leaks were discarded. Subsequently, temperature was raised to 37°C and arteries were allowed to equilibrate at 70 mmHg (in the absence of intraluminal flow) to develop MT manifested as ∼20% decrease in diameter. Only arteries that exhibited spontaneous and stable MT were studied. Vascular viability was confirmed in all arteries by vasoconstrictor responses to phenylephrine (PE) and vasodilator responses to ACh. Where required, endothelium was denuded by passing an air bubble through the arteries. Arteries were allowed to stabilize for 20–30 min. Denudation was confirmed by loss of response to ACh.

### Diameter measurement

Arteries were viewed with a 10× objective on a Nikon TMS microscope equipped with a monochrome video charge-coupled device camera. The luminal diameter was measured by image capture with a video frame grabber and real-time edge-detection (Living Systems; Burlington, VT or Ionoptix, Milton, MA). Data were continuously sampled and recorded with Axoscope software (Axon Instruments or Ionoptix, Milton, MA). Passive diameter (PD) was determined at the end of each experiment by incubating arteries in Ca^2+^-free PSS for 20 min.

### Calcium fluorescence

In experiments where a Ca^2+^ indicator was used, the selected artery was exposed to dissection solution containing fluo-2 (TEFLabs, 7.5 µM), 1.5% DMSO (vol/vol), and 0.03% cremophor EL (vol/vol) for 1 h at room temperature. After 1 h, the arteries were cannulated as described above and allowed to develop MT for 30 min. Arteries were imaged with a confocal scanning inverted microscope (×60, 1.4 NA, water-immersion objective). Images of fluo-2-loaded fluorescent VSM were obtained with an intensified CCD camera (Stanford Photonics, Palo Alto, CA, USA) coupled to a Nipkow spinning disk confocal microscope with 488 nm excitation. Spatially resolved information on cytoplasmic [Ca^2+^] was obtained in individual VSM cells. To quantify changes, fluo-2 fluorescence (*F*) was normalized to its initial value (*F*_0_) in each cell.

### Myogenic response

To assess myogenic response, arteries were subjected to incremental pressures between 20 and 100 mmHg (20, 40, 60, 80 and 100). At each pressure, spontaneous MT was allowed to develop until a stable diameter was achieved, usually within 5 min. After completion of the pressure-response curve, intraluminal pressure was maintained at 20 mmHg, and arteries were superfused with Ca^2+^-free PSS with EGTA 3 mM plus diltiazem 0.01 mM. The pressure-response curve was then repeated to measure the corresponding passive diameters. MT was calculated as the percent difference in diameter observed for Ca^2+^-containing versus Ca^2+^-free PSS at each pressure.

### RNA extraction and quantitative PCR (qPCR)

Small arteries parallel to the small intestine were dissected, pooled and stored in 1 ml Trizol® Reagent (Invitrogen). RNA was extracted from tissue homogenates using standard isolation procedures and stored at −80°C. After DNase treatment for 30 min, RNA was subjected to first strand cDNA synthesis with random hexamer primers using the Protoscript® II RT-PCR kit (New England Biolabs, Ipswich, MA). cDNA (1 µl) was amplified for 30 cycles using 2xPCR buffer (25 µl), Taq polymerase (1 µl) and primers (Table 1) designed to amplify the target sequences. Primers were designed to span an intron and all qPCR products were of the predicted size. qPCR was carried out in a Perkin Elmer Gene Amp PCR System 9700 block (Perkin-Elmer, Norwalk, CT U.S.A). One microliter of autoclaved RNAse- and DNase-free water was amplified to control for nucleic acid contamination. After the first-round PCR, the amplified product (1 µl) was further subjected to nested PCR for 30 cycles using a second set of primers (Table 2). Control (10 µl) and nested-reaction products (10 µl) were subjected to electrophoresis on a 1.0% agarose gel containing 0.001% EtBr and visualized by fluorescent illumination (BioRad, Hercules, CA).

**Table 1:** First-round PCR Primer Sequences.

Receptor subtype	Primer sequence	PCR product (bp)
M_1_R forward	gcacaggcacccaccaagcag	373
M_1_R reverse	agagcagcagcaggcggaacg	
M_2_R forward	ggcaagcaagagtagaataaa	552
M_2_R reverse	gccaacaggatagccaagatt	
M_3_R forward	gtctggcttgggtcatctcct	434
M_3_R reverse	gctgctgctgtggtcttggtc	
M_4_R forward	agtgcttcatccagttcttgtcca	588
M_4_R reverse	cacattcattgcctgtctgctttg	
M_5_R forward	ctcatcattggcatcttctcca	394
M_5_R reverse	ggtccttggttcgcttctctgt	
β-actin forward	gtttagaaccttcaacacc	354
β-actin reverse	gtggccatcctcttgctcgaagt	

bp-base pair.

**Table 2:** Second-round PCR Primer Sequences.

Receptor subtype	Primer sequence	PCR product (bp)
M_1_R forward	cctcccaaaagctcccca	331
M_1_R reverse	tgtcccggaaggctttgt	
M_2_R forward	gaaggaaaagaaggaacctgt	510
M_2_R reverse	gtcctggtcactttcttttcc	
M_3_R forward	cctatgggctcctgccat	388
M_3_R reverse	ttggtccatctgctcggca	
M_4_R forward	acccggcagtgacctttg	540
M_4_R reverse	ttacaatttgaatcttggaccat	
M_5_R forward	gaacctctacacgacctacatc	350
M_5_R reverse	cccggtagatccggcagtag	
β-actin forward	aaccgcgagaagatgacccagatcatgttt	333
β-actin reverse	agcagccgtggccatcttgctcgaagtc	

bp-base pair.

### Reagents

DCG and ACh were prepared from stock solution by serial dilutions into freshly prepared PSS. To test for roles of muscarinic receptors, arteries were incubated with inhibitors for at least 30 min before adding DCG and ACh. Muscarinic receptor antagonists included pirenzepine (1 µM, M_1_R), methoctramine (1 µM, M_2_R), 4-DAMP (0.1 µM, M_3_R), tropicamide (1 µM, M_3,4_R) and atropine (1 µM). Experiments to assess non-PGI_2_ and non-NO vasodilation were performed by pre-incubating arteries in indomethacin (INDO, 10 µM) and L-NAME (300 µM) for at least 40 min. Experiments to assess the effects of K^+^ channel subtype blockade employed 4-aminopyridine (4-AP; 1 mM, K_V_-inhibitor), BaCl_2_ (30 µM; K_IR_-inhibitor), glibenclamide (10 µM; K_ATP_-inhibitor) and tetraethylammonium (TEA 10 mM; K_Ca_-inhibitor). All reagents and inhibitors were purchased from Sigma Aldrich.

### Data handling and statistical Analysis

DCG and ACh effects were observed for at least 5 min. The diameter of arteries undergoing vasomotion was calculated by averaging the plateau phase for 1 min. The data are expressed as percent of maximal relaxation (%Max) according to the relationship: %Max = 100× [ΔD_C_/ΔD_B_]; where ΔD_B_ is the difference between PD in Ca^2+^-free buffer and basal diameter and ΔD_c_ is the difference between diameter in DCG or ACh and basal diameter.

Myogenic response (MR) is expressed as a percent of PD, and calculated from the basal diameter as : 100× (PD–basal diameter)/PD.

Comparison of means was performed using Student's *t*-test (two groups) or ANOVA (multiple groups) as appropriate. Results are presented as mean ± S.E.M. Three to 12 rats were used for each experimental condition; in the figure legends n represents the number of arteries examined. Significance was defined as *p*<0.05 and expressed in illustrations as ^*^ *p*<0.05, ^**^ *p*<0.01, and ^***^ *p*<0.001.

## Supporting Information

Figure S1 **Effect of 4-DAMP on myogenic response.** Fourth-order rat mesenteric arteries pretreated with vehicle and 0.1 µM 4-DAMP were subjected to a series of intraluminal pressure steps between 20 and 100 mmHg and spontaneous tone was allowed to develop until a stable diameter was achieved. The pressure-response was repeated in Ca^2+^-free physiological salt solution (PSS) with 3 mM EGTA and 0.01 mM diltiazem. MT was calculated as the percent difference in diameter observed for Ca^2+^-containing vs. Ca^2+^-free PSS at each pressure. 4-DAMP (0.1 µM), which inhibits ACh-induced reduction of MT, had no effect on myogenic response. (n = 4 arteries in each group).
(DOC)

Figure S2 **Role of cGMP in DCG-induced reduction of MT in rat 4^th^-order mesenteric arteries.** To determine the role of VSM guanylyl cyclase, a downstream effector of NO, we examined the effect of ODQ (10 µM), a guanylyl cyclase inhibitor, on DCG (100 µM)-induced reduction of MT. All experiments were conducted in the presence of 300 µM L-NAME and 10 µM INDO. Incubation with ODQ did not alter DCG-mediated reduction of MT. (n = 3 arteries in each group).
(DOC)
