# Supplementary material for: Effects of Deoxycholylglycine, a Conjugated Secondary Bile Acid, on Myogenic Tone and Agonist-Induced Contraction in Rat Resistance Arteries
Source: PLoS One. 2012 Feb 16;7(2):e32006. doi: 10.1371/journal.pone.0032006 (PMC3281111; doi:10.1371/journal.pone.0032006)
Supplement: Figure S1 — Effect of 4-DAMP on myogenic response. Fourth-order rat mesenteric arteries pretreated with vehicle and 0.1 µM 4-DAMP were subjected to a series of intraluminal pressure steps between 20 and 100 mmHg and spontaneous tone was allowed to develop until a stable diameter was achieved. The pressure-response was repeated in Ca2+-free physiological salt solution (PSS) with 3 mM EGTA and 0.01 mM diltiazem. MT was calculated as the percent difference in diameter observed for Ca2+-containing vs. Ca2+-free PSS at each pressure. 4-DAMP (0.1 µM), which inhibits ACh-induced reduction of MT, had no effect on myogenic response. (n = 4 arteries in each group). (DOC) [file pone.0032006.s001.doc]

**Supplementary Information 1**

**Figure S1. Effect of 4-DAMP on myogenic** **response.** Fourth-order rat mesenteric arteries **pretreated with vehicle and 0.1** µM **4-DAMP** were subjected to a series of intraluminal pressure steps between 20 and 100 mmHg and spontaneous tone was allowed to develop until a stable diameter was achieved. The pressure-response was repeated in Ca^2+^-free physiological salt solution (PSS) with 3 mM EGTA and 0.01 mM diltiazem. MT was calculated as the percent difference in diameter observed for Ca^2+^-containing vs. Ca^2+^-free PSS at each pressure. **4-DAMP (0.1** µM**), which inhibits ACh-induced reduction of MT, had no effect on myogenic response**. (n = 4 arteries in each group).
