# Supplementary material for: Effects of Deoxycholylglycine, a Conjugated Secondary Bile Acid, on Myogenic Tone and Agonist-Induced Contraction in Rat Resistance Arteries
Source: PLoS One. 2012 Feb 16;7(2):e32006. doi: 10.1371/journal.pone.0032006 (PMC3281111; doi:10.1371/journal.pone.0032006)
Supplement: Figure S2 — Role of cGMP in DCG-induced reduction of MT in rat 4th-order mesenteric arteries. To determine the role of VSM guanylyl cyclase, a downstream effector of NO, we examined the effect of ODQ (10 µM), a guanylyl cyclase inhibitor, on DCG (100 µM)-induced reduction of MT. All experiments were conducted in the presence of 300 µM L-NAME and 10 µM INDO. Incubation with ODQ did not alter DCG-mediated reduction of MT. (n = 3 arteries in each group). (DOC) [file pone.0032006.s002.doc]

**Supplementary Information 2**

**Figure S2. Role of cGMP in DCG-induced reduction of MT in rat 4^th^-order mesenteric arteries.** To determine the role of VSM guanylyl cyclase, a downstream effector of NO, we examined the effect of ODQ (10 µM), a guanylyl cyclase inhibitor, on DCG (100 µM)-induced reduction of MT. All experiments were conducted in the presence of 300 µM L-NAME and 10 µM INDO. Incubation with ODQ did not alter DCG-mediated reduction of MT. (n = 3 arteries in each group).
